# Synthetic aircraft RS image modelling based on improved conditional GAN joint embedding network

**DOI:** 10.1038/s41598-021-03880-x

**Published:** 2022-01-10

**Authors:** Junyu Chen, Haiwei Li, Liyao Song, Geng Zhang, Bingliang Hu, Shuang Wang, Song Liu, Siyuan Li, Tieqiao Chen, Jia Liu

**Affiliations:** 1 Key Laboratory of Spectral Imaging Technology of Chinese Academy of Sciences, Xi’an Institute of Optics and Precision Mechanics of CAS, Xi’an, 710119 China; 2 University of Chinese Academy of Sciences, Beijing, 100049 China; 3 School of Information and Communications Engineering, Xi’an Jiaotong University, Xi’an, 710049 China

**Keywords:** Electrical and electronic engineering, Aerospace engineering, Information technology

## Abstract

Developing an efficient and quality remote sensing (RS) technology using volume and efficient modelling in different aircraft RS images is challenging. Generative models serve as a natural and convenient simulation method. Because aircraft types belong to the fine class under the rough class, the issue of feature entanglement may occur while modelling multiple aircraft classes. Our solution to this issue was a novel first-generation realistic aircraft type simulation system (ATSS-1) based on the RS images. It realised fine modelling of the seven aircraft types based on a real scene by establishing an adaptive weighted conditional attention generative adversarial network and joint geospatial embedding (GE) network. An adaptive weighted conditional batch normalisation attention block solved the subclass entanglement by reassigning the intra-class-wise characteristic responses. Subsequently, an asymmetric residual self-attention module was developed by establishing a remote region asymmetric relationship for mining the finer potential spatial representation. The mapping relationship between the input RS scene and the potential space of the generated samples was explored through the GE network construction that used the selected prior distribution z, as an intermediate representation. A public RS dataset (OPT-Aircraft_V1.0) and two public datasets (MNIST and Fashion-MNIST) were used for simulation model testing. The results demonstrated the effectiveness of ATSS-1, promoting further development of realistic automatic RS simulation.

## Introduction

Modelling and simulation (M&S) in the RS domain is cr ucial for instrument design, algorithm development, data classification, target recognition, data augmentation, and disaster warning^1^. With the high timeliness and high-quality demand for the RS technology, efficient and higher quality simulations have become a critical requirement^2^.

Several M&S methods in the RS domain have been demonstrated in the literature. Ba sed on the technical route, the RS target modelling can be divided into two types: three-dimensional (3D) modelling^3^ and deep learning (DL) modelling^4^.

3D models such as SENSOR^5^, DIRSIG^6^, or FASSP^7^ depend on modelling tools such as AutoCAD or 3DsMAX to construct a 3D surface model, which leads to inconvenience and lower efficiency. As a result, these methods limit the requirements of the RS applications for volume, velocity, variety, and value (4V).

DL modelling employs the generative adversarial network (GAN) technology^8^ to learn the potential space distribution by training data samples. For example, CGAN^9^ constructs a condition generator by simply entering condition label y. ACGAN^10^ extends from CGAN, which realises the function of multi-type image generation and classification by introducing an auxiliary classifier after the discriminator and a deep convolutional neural network (DCNN)^10,11^ as its underlying architecture. However, since it puts the ground-truth samples and generated samples into one classifier to judge together, the pattern collapse is easy to occur on intra-class. Later, based on CGAN, several variants (e.g., styleGAN^12^, GauGAN^13^, OR-AC-GAN^14^, improved GAN^15^.) appeared in succession and were applied to the face domain, natural scene, medical simulation, hyperspectral anomaly detection, and other fields. In this way, we could construct the rich and diverse generated targets through potential spatial representation. Past literature focuses on RS-scene modelling by augmenting data samples to carry out corresponding tasks, such as scene classification, scene recognition. For example, in^16^, Attention-GANs were utilised to extend samples on public RS classification datasets and were then applied to aerial scene classification. As a preliminary attempt, this paper provides a theoretical basis that applies the GAN to the RS-image modelling.

With the growing application requirements, it becomes an urgent task to carry out a precise M&S in a subclass field (fine-grained data). For example, aircraft has a profound theoretical significance and substantial application value . If the DL technology achieves low-cost 4V simulation for different RS aircraft types, it will have a significant impact on academia and industry^4^.

However, due to several uncertain factors such as altitude , illumination, occlusion, and background interference, there are slight inter-class differences and significant intra-class differences. Compared to ordinary generative tasks, fine-grained image generation is more complicated. It is easier to classify different subclass into the same category because of the short distance between the spatial features of similar sub-classes when using a convolutional neural network (CNN)^17^ or DCNN^10,11^ in the generator. As a result, different aircraft types generate the same style, resulting in occurrences of feature entanglement between sub-classes. Reviewing the past tasks of subcategory feature extraction, relevant scholars used manual labelling (e.g., bounding box, part locations), local feature extraction (e.g., scale-invariant feature transform^18^, histograms of oriented gradient^19^), and CNN joint class labels to extract feature. With the development of these methods, the accuracy is constantly improving. Moreover, some scholars introduced attention mechanisms into the networks^20^, which redistribute the available information and focus on the salient components for the input data. In this case, SAGAN^21^ combines the self-attention block in the network and minimises the hinge version of the adversarial loss from the support vector machine (SVM)^22,23^ to improve the generation accuracy. Conditional Batch Normalisation (CBN)^24–26^ is proposed for the recalibration of different class-wise feature responses by conducting internal normalisation on the same category of data feature graphs. However, it ignores the characteristics of the spatial location by using conditional information statistics in the CBN process, suppressing the generation of different categories to some extent. Therefore, more acceptable simulation methods need to be studied urgently.

The development of GAN makes it possible to build 4V models for RS targets. However, a GAN cannot express the mapping relationship between the ground-truth scene and potential space when constructing the specified target from an RS scenario. It involves the pre-network mapping problem of the GAN, i.e., GAN-embedding. In general, there are two methods to embed the input samples into the potential space. 1. Learn an encoder such as an auto-encoder (AE) or a variational auto-encoder (VAE) that maps a given image to a potential space^27^. 2. Select a random initialisation prior vector and optimise it by using gradient descent^28,29^. Among these, the first approach provides a quick solution for image embedding by performing forward transmission through the encoder. However, it often extends beyond the training dataset. In this article, we decided to reference the second approach as a more general and stable solution.

According to the above research, there are three main problems in the fine modelling of different aircraft: 1. The feature entanglement in fine-grained target modelling when using a GAN; 2. More delicate texture modelling; 3. Optimisation of the mapping relationship between a simulated RS aircraft and RS scene.

To address these issues, we utilise a novel synthetic aircraft RS-image modelling technology, the first-generation highly realistic aircraft type simulation system (ATSS-1), which is based on an improved GAN module in combination with an embedding network. The experiment is conducted on a public RS dataset of OPT-Aircraft _V1.0^30^, which consists of 14 global aircraft types such as flat-wing, rear-wing, and propeller type. The main contributions can be summarised as follows:

1. In ATSS-1, a novel GAN model (an adaptive weighted conditional attention generative adversarial network, AWCA-GAN) is integrated to an improved embedding network (geospatial embedding network, GE) to form one system, correlates with real RS scene, and simulates RS data without complex manual manipulation.
2. An adaptive weighted conditional batch normalisation attention mechanism module (AWCBNA) in AWCA-GAN is presented, applying intra-class-weighted feature statistics to recalibrate category feature responses adaptively for the conditional parameters. Such AWCBNA block enables the network to emphasise the category features selectively and enhances the ability of presentation learning in different types to alleviate the entanglement of subclass features.
3. An asymmetric residual self-attention module (ARSA) is an add-on to the AWCA-GAN that captures the spatial geometry and spectral information by establishing a remote region asymmetric relationship to obtain a more robust feature representation.
4. An efficient embedding network, called a geospatial embedding network (GE), is investigated, which could map a real RS scene into a latent space of the simulated target, which is drawn from prior distribution z as the intermediate representation through the trained AWCA-GAN model.
5. In the experiment, we collect aircraft datasets (namely, OPT-Aircraft _V1.0^30^) and investigate the effect of aircraft RS-image simulation on high-level visual tasks . This dataset is a fine-grained public dataset of aircraft in the RS field. It can provide benchmark data for future research in RS fine-grained identification, classification and processing.

## Methods

**Figure 1:**
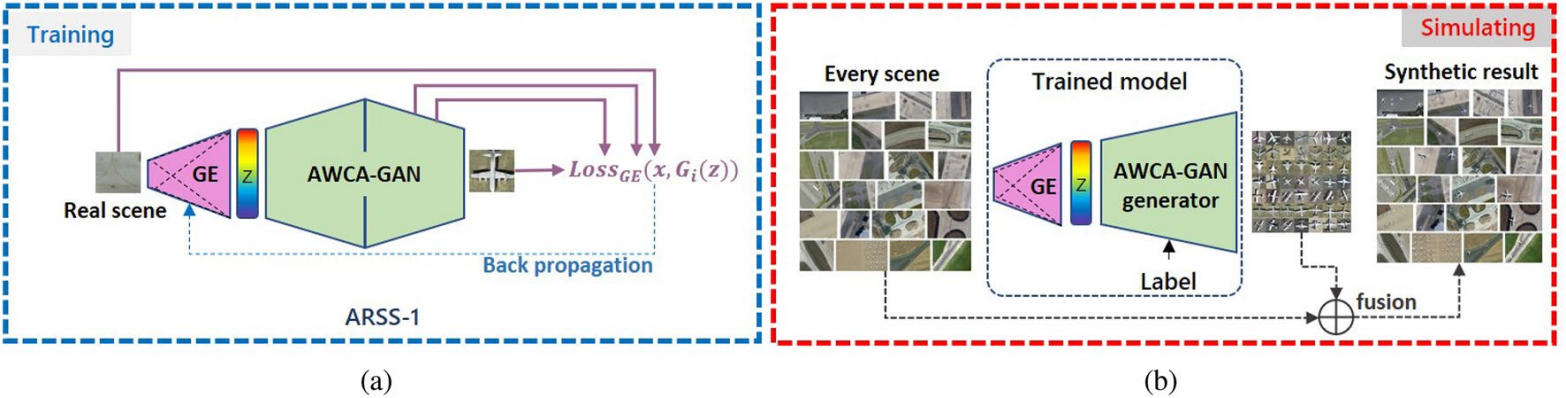
Overall workflow of our ATSS-1. (**a**) Two sub-networks in ATSS-1 and the training process. (**b**) Diagram of simulating process. When performing a simulating task, each sample is put into the trained ATSS-1 to generate the required images and then applying the post-processing step of Poisson blending^31^ to synthesise the final RS images.

Figure 1 depicts the overall workflow of our ATSS-1. The scheme starts from the RS aircraft datasets: First, we use an improved conditional GAN to learn the distribution spaces of multiple aircraft type samples from random distributions such as Gaussian or uniform distribution. Then, we use random vectors with labels as intermediaries to calculate the transformation space from the region to be simulated to the RS target. Finally, we apply the Poisson blending to synthesise the final RS images. The core part mainly includes two sub-networks. 1) RS-image modelling AWCA-GAN, 2) embedding network GE. For the first part, RS-image modelling utilises AWCA-GAN to achieve more elaboration latent space representation for different RS-image types. The second sub-network GE extracts an optimizer mapping from input real RS scene to latent space. Because of these two sub-networks, the potential space of generative network and features of input real RS scene associated.

### Simulated images modelling: AWCA-GAN

**Figure 2:**
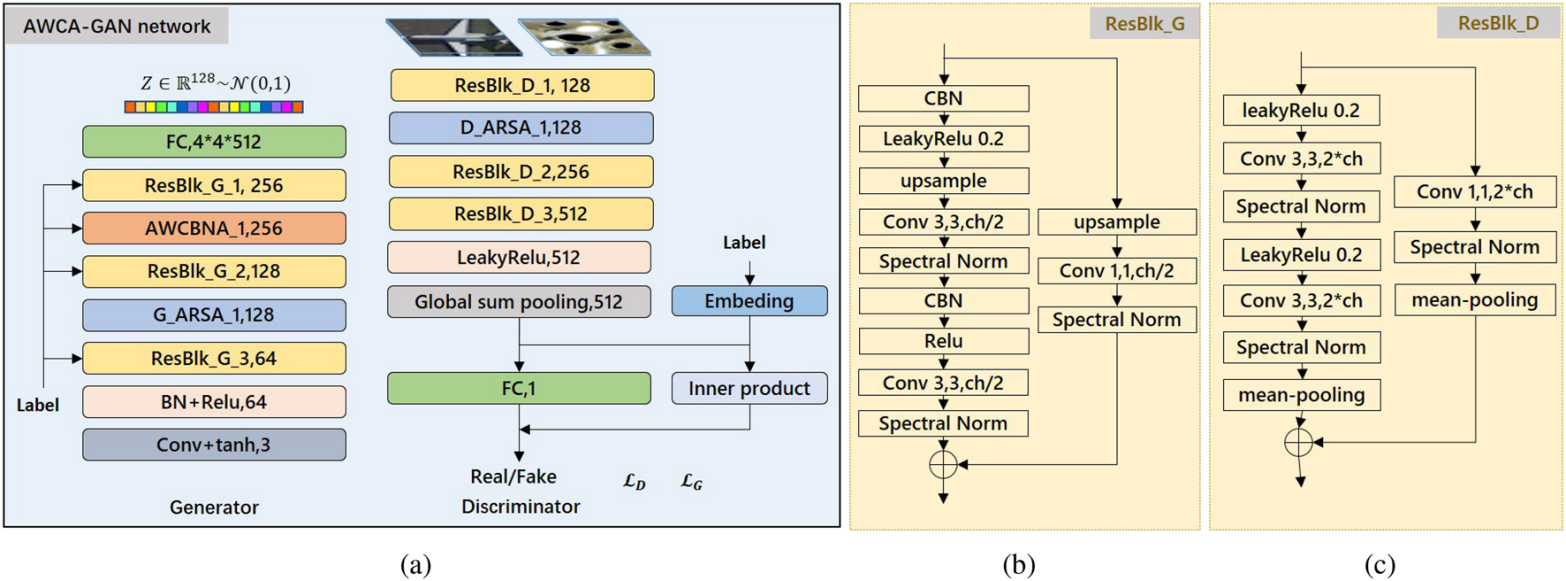
Structure details of AWCA-GAN. (**a**) The architecture of AWCA-GAN. (**b**) Residual block of the generator (ResBlk_G) in AWCA-GAN. (**c**) Residual block of the discriminator (ResBlk_D) in AWCA-GAN.

The specific network structure of the AWCA-GAN illustrated in Fig. 2, consists of a discriminator and a generator. In the AWCA-GAN generator, a fully connected layer is fed to extract the potential characteristics from the input. Then, three residual modules (ResBlk_G_1 to ResBlk_G_3) with global spectral normalisation (SN) layers^32^ are stacked to relieve the gradient vanishing and mode collapse. Next, AWCBNA is added after ResBlk_G_1 to reallocate the inter-class-wise feature responses adaptively. After that, one ARSA module in the generator is employed to obtain a more robust feature representation by establishing the remote regional relationships to extract the global geometric features. Thereafter, we use the ReLU activation function to optimise the generator convergence to the global optimum due to its advantages of unilateral inhibition, relatively wide excitation boundary, and sparse activation except in the final layer of the generator. At the same time, we use a convolutional layer with the tanh activation function to constrain the output ranges from -1–1 to ensure that the data range is consistent with the training set when it is sent to the discriminator. Furthermore, to introduce more significant nonlinearity and accelerated convergence into the discriminator, we select the Leaky-ReLU instead of the ReLU as the activation function to keep the training process in Nash equilibrium and set the leaky value equal to 0.2 is empirically obtained for good performance. Similar to cGANs of projection discriminator^24^, AWCA-GAN uses projection in the discriminator. Figure 2b and c for more detail on ResBlk_G and ResBlk_D. Finally, we use the hinge loss^21,22^ of the standard conditional adversarial loss to guide the network training effectively.

#### Adaptive weighted conditional batch normalization attention block: AWCBNA

To develop a more robust learning ability of category feature generation, an AWCBNA module is developed to unmix the feature of inter-class by exploring adaptive category-weighted conditional batch normalization statistics.

**Figure 3:**
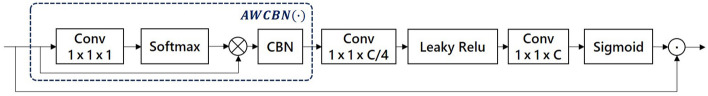
Network framework of adaptive weighted conditional batch normalisation attention module (AWCBNA).

Figure 3 shows an intermediate data cube $F \in {\mathbb {R}}^{W \times H \times C}$ being fed into a $1\times 1\times 1$ convolutional kernel to learn the spatial adaptive weighted matrix $A \in {\mathbb {R}}^{W \times H \times 1}$. Then, an activation function (Softmax) is applied to normalise A and multiply F with A to get adaptive weighted channel-wise feature map $M \in {\mathbb {R}}^{C \times 1}$. To make full use of the class condition information in M, we adopt CBN^24–26^ to normalise the set of adaptive weighted channel-wise feature map M from the batch by a pair of class-specific scale and bias parameters. We define the above procedure as adaptive weighted conditional batch normalisation:

1 $$\begin{aligned} {\text {AWCBN}}(M)=\gamma (c) \frac{M-E[M]}{\sqrt{{\text {Var}}[M]+\epsilon }}+\beta (c) \end{aligned}$$

where $\gamma (c)$, $\beta (c)$ are the trainable scale and bias parameters specific to the *c* class, respectively.

To further optimise the inter-class features, we adopt a simple strategy of applying two convolutional layers with 1$\times$1$\times$C/4 kernel, 1$\times$1$\times$C kernel, and activation functions (Leaky-ReLU and Sigmoid). As a result, we obtain channel attention *h*(*F*) and then combine the channel attention *h*(*F*) and parameter $\zeta$ to rescale the intermediate data cube F.

2 $$\begin{aligned} {\hat{F}}=F+\zeta h(F) F \end{aligned}$$

where *h*(*F*) and $\zeta$ are the scaling factors, $\zeta$ initialise to 0. Through the training of *h*(*F*), $\zeta$, and the operating factor of ${\text {AWCBN}}(\cdot )$, ${\text {AWCBN}}$ could recalibrate inter-class-wise characteristics to boost the conditional generated ability.

#### Asymmetric residual self attention block: ARSA

**Figure 4:**
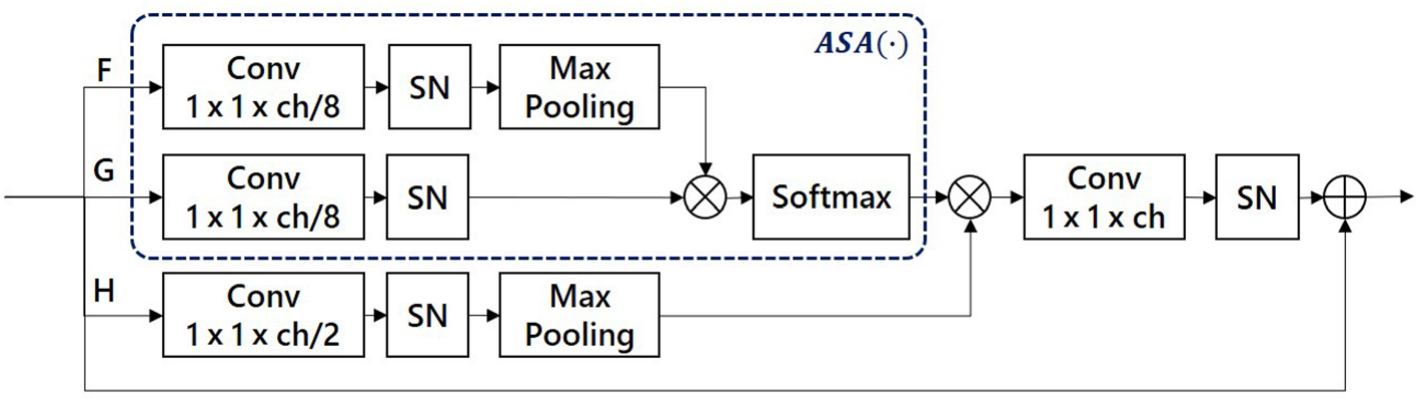
Detailed architecture structure of ARSA block. The blue dotted box represents the process of ${\text {ASA}}(\cdot )$.

To achieve a more power feature-related learning, we design an ARSA block based on a self-attention mechanism^21^, which retains meaningful long-distance features and suppresses interference information by establishing asymmetric global regional relations. The specific structure is shown in Fig. 4.

Just like self-attention^21^, it contains three branches: F-branch $f\left( x_{i}\right)$, G-branch $g\left( x_{i}\right)$, and H-branch $h\left( x_{i}\right)$. $f\left( x_{i}\right)$ and $g\left( x_{i}\right)$ are used to calculate the attention level of the spatial position $f(x)={\text {SN}}\left( W_{f} * x\right)$ and $g(x)={\text {SN}}\left( W_{g} * x\right)$, ${\text {SN}}(\cdot )$ represents SN^32^. To obtain more optimised spatial characteristic parameters, before F-branch and G-branch are multiplied, F-channel is subsampled. Thereafter, an activation function (Softmax) is used to obtain different attention levels. We define the above process as asymmetric spatial attention ASA($\cdot$):

3 $$\begin{aligned} {\text {ASA}}\left( x_{i}\right) =\frac{\exp \left( {\text {Down}}\left( f\left( x_{i}\right) \right) ^{T} \times g\left( x_{i}\right) \right) }{\sum _{i=1}^{N} \exp \left( {\text {Down}}\left( f\left( x_{i}\right) \right) ^{T} \times g\left( x_{i}\right) \right) } \end{aligned}$$

where $x_{i}$ represents the feature cube entering the ARSA block; ${\text {Down}}(\cdot )$ represents the max-pooling.

Integrate global spatial information and local information $h(x)={\text {Down}}\left( {\text {SN}}\left( W_{h} * x\right) \right)$ through $h\left( x_{i}\right)$. Finally, the asymmetric attention map is mapped to the input channel through a convolutional layer with a kernel size of 1*1. The output result obtains by combining the input data cube x and parameter $\gamma$. $y=x+\gamma \times v\left( \sum _{i=1}^{N} A {\text {SA}}\left( x_{i}\right) h\left( x_{i}\right) \right)$. Where $\gamma$ is a learnable scalar that initialises as 0 and allocates more weight to the ASA map piecemeal according to the global feature in the training process.

### Geospatial embedding network: GE

This section focuses on the process of mapping from an input sample $I\in {\mathbb {R}}^{n \times n \times 3}$ to the latent space (i.e., generated image $G\left( z^{*}, y\right)$) on trained AWCA-GAN. If the prior distribution $z^{*} \in {\mathbb {R}}^{n}$ is taken as the intermediate feature representation to establish the projection relationship, it can be reduced to the following minimisation problem: $z^{*}=\min _{z}-{\mathbb {E}}_{x} D(G(z, y), y)$.

GE follows a direct forward optimisation framework embedding a given sample into the manifold projection of a pre-trained generator. Acting from a feasible initial latent vector z, the search for an optimised vector z* by using gradient descent to minimise the loss function that monitors the semantic and multiple-level perceptual diversities between the feature of inputting samples and simulated RS data $G\left( z^{*}, y\right)$ from $z^{*} \in {\mathbb {R}}^{n}$. For the loss function, we define three weighted combination loss functions of style loss ${\mathscr {L}}_{\text{ style } }$, type loss, and pixel-wise MSE loss:

4 $$\begin{aligned} \begin{aligned} {\mathscr {L}}_{z^{*}}=\min _{z^{*}} \alpha {\mathscr {L}}_{\text{ style } }\left( G\left( z^{*}, y\right) , I\right) +\beta {\mathbb {E}}_{I} D\left( G\left( z^{*}, y\right) , y\right) +\frac{\gamma }{N}\left\| G\left( z^{*}, y\right) -I\right\| _{2}^{2} \end{aligned} \end{aligned}$$

where $I\in {\mathbb {R}}^{n \times n \times 3}$ is the pre-selected inputting sample, ${\text {G}}(\cdot )$ is the trained generator of AWCA-GAN, N is the number of scalars in the input samples, $z^*$ is a prior vector to be optimised. $\alpha$, $\beta$, and $\gamma$ are controllable hyperparameters. $\alpha = 0.4$, $\beta =0.2$, and $\gamma =0.4$ are empirically obtained for good performance.

In Eq. 4, the first, second,and third term are style loss, type reconstruction loss, and pixel loss, respectively. The type reconstruction loss measures the difference between the generated image by $z^{*}$ and the pre-set type, which calculates the minimum value in the final feature space of the trained discriminator. Pixel loss is the (normalized) Euclidean distance between the generated image $G\left( z^{*}, y\right)$ and the input samples *I*. The style loss term ${\mathscr {L}}_{\text{ style } }(\cdot )$ penalises the differences in style: radiation, textures, etc.

5 $$\begin{aligned} \begin{aligned} {\mathscr {L}}_{\text {style}}\left( x_{1}, x_{2}\right) =\sum _{i=1}^{3} \frac{\lambda _{i}}{N_{i}}\left\| {\text {Gram}}\left( D_{i} \left( x_{1}\right) \right) -{\text {Gram}}\left( D_{i}\left( x_{2} \right) \right) \right\| _{F}^{2} \end{aligned} \end{aligned}$$

where $x_{1}, x_{2} \in {\mathbb {R}}^{n \times n \times 3}$ are the input two samples, ${\text {D}}(\cdot )$ is the feature map output of the trained discriminator layers ResBlk_D_1, ResBlk_D_2, and ResBlk_D_3 respectively, $N_{i}$ is the number of scalars in the i-th layer output data cube, $\lambda _{i}$ is controllable hyperparameters for i-th layer, and $\lambda _{i}=1$ are empirically obtained for good performance.

## Related work

### Dataset and evaluation metrics

Datasets To experimentally verify the algorithm’s effectiveness on the RS images, we collected an RS dataset (namely OPT-Aircraft_V1.0) and made them available in the literature^30^, which contains 14 aircraft types for remote sensing globally, with a total number of 3,594 and a size of 96 $\times$ 96 $\times$ 3. However, some aircraft types are not suitable for the study of generating models due to their small number of samples. Considering this, we choose seven aircraft types, including 656 Swept-back wing aircraft I, 320 Swept-back wing aircraft III, 75 Swept-back aircraft with leading-edge II, 192 Delta-wing aircraft, 1088 Flat-wing aircraft II, 414 Propeller aircraft II, 242 Propeller aircraft III, with a large number of samples to guide the learning of the proposed algorithm. In addition, we augmented the data in the dataset. We performed rotations of the data by $90^\circ$, $180^\circ$, and $270^\circ$, mirror image flipping, up-and-down flipping, and seven other operations to keep the subclass samples balanced and obtained a total of 30,464 aircraft RS images. Select 90% of the aircraft samples randomly as a training set and 10% of the aircraft samples as a testing set. The training set of aircraft is named OPT_Aircraft-7. All the data was limited to 32 $\times$ 32 $\times$ 3. Besides, to verify the robustness of the model, we selected MNIST^33^, Fashion-MNIST^34^ as a more detailed illustration.

Evaluation metrics One metric index used in Fréchet Inception Distance (FID) index^35^ to evaluate the performance of the generative model. A smaller FID indicates a better generative model.

6 $$\begin{aligned} {\text {FID}}=\left\| \mu _{\text{ data } }-\mu _{g}\right\| +{\text {tr}}\left( \sum _{\text{ data } }+\sum _{g}-2\left( \sum _{\text{ data } } \sum _{g}\right) ^{\frac{1}{2}}\right) \end{aligned}$$

where $\mu _{g}$ and $\sum _{g}$ represent the mean and variance of the generative samples, and $\mu _{data }$ and $\sum _{data}$ represent the mean and variance of the training samples, respectively. In this experiment, we used Inception-v3 as the classifier and used 1,000 dimensions of the penultimate layer of the network as the feature layer.

### Implementation detail

1. Experimental configuration: The experiment framework used was Python 3.6, the programming language was TensorFlow 1.14.0, and the computer operating environment was Ubuntu 18.04.3. The computer processor was an Intel Xeon®E5-2678 V3; the graphics card was a GeForce GTX 1080ti; the memory was 64 GB.
2. Image modelling: To prove the quality of the generated data, we fed MNIST^33^, Fashion-MNIST^34^, and OPT_Aircraft-7^30^ datasets into AWCA-GAN. We trained the AWCA-GAN from scratch. In order to ensure more stable training of the generated model, we set an imbalanced learning rate according to reference^35^. The initial learning rate of the generator was 0.0001, and that of the discriminator was 0.0004. The maximum training epochs set at 30,000. The learning rate fell to 80% with every 300 training epochs. The entire process guided the generator towards the global optimum. At this learning rate setting, we observed no significant jump in sample quality or FID value during training. The loss of generator and discriminator keep in Nash equilibrium. Because of the Leaky-ReLU activation function, the Xavier initialisation method was applied for the network parameters.
3. Embedding modelling: To analyse the potential association between the RS scene and the generated images by the intermediate prior variable $z^{*} \in {\mathbb {R}}^{128} \sim {\mathscr {N}}(0,1)$, we selected a batch of pre-selected RS scene from the DIOR^36^, UCAS_AOD^37^, NWPU_VHR-10^38–40^, DOTA^41^ dataset and high-resolution images in Google Earth and fed a $32 \times 32$ embeddable region to the GE network by minimising the loss function described by Eq. 4. In the GE experiment, we used Adam optimiser to train the prior variable $z^*$ for 5000 iterations with a learning rate of 0.001. An outstanding embedding process should that an optimised prior vector $z^{*}$ map to a desired simulated sample $G\left( z^{*}, y\right)$. In this way, it produces an image $G\left( z^{*}, y\right)$ that could learn several high-level semantic information from the input RS scene when passed through the trained generator.

### Visual examples of AWCA-GAN

We conducted experiments on MNIST, Fashion-MNIST and OPT_Aircraft-7 datasets to verify the AWCA-GAN algorithm’s effectiveness.

**Figure 5:**
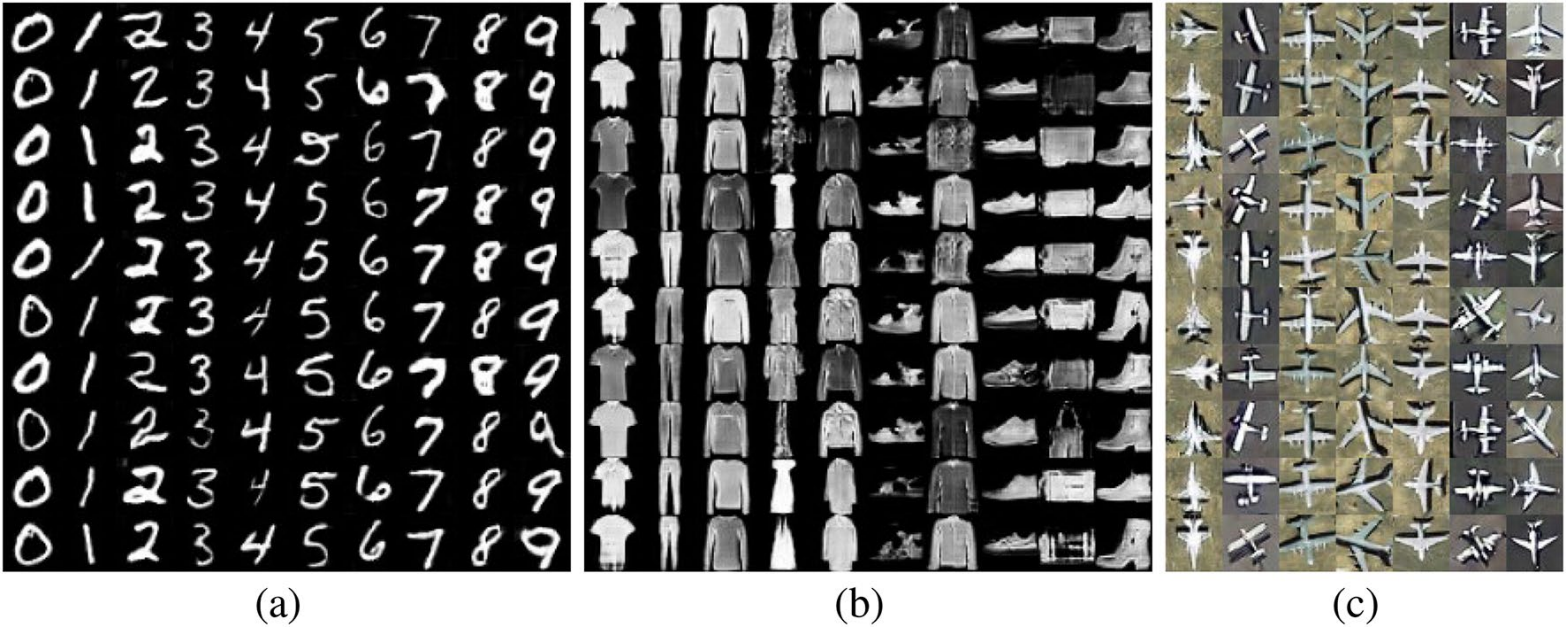
Examples of generated samples of AWCA-GAN on three datasets: (**a**) OPT_Aircraft-7; (**b**) MNIST; and (**c**) Fashion-MNIST.

As shown in Fig. 5, the images generated by AWCA-GAN were closer to the ground-truth images and provided complete texture in the geometric structure. As a result, a more precise outline obtained between the target and background, and the generated samples were more dynamic, structural and diverse.

**Figure 6:**
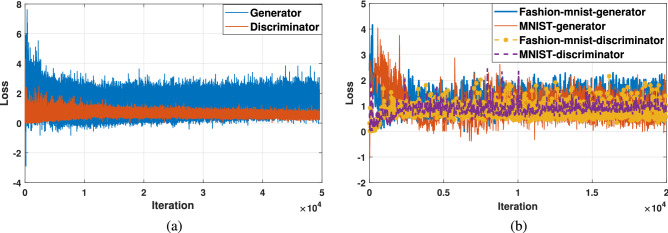
Comparison of AWCA-GAN loss on three datasets of OPT_Aircraft-7, MNIST and Fashion-MNIST over training stages. (**a**) Loss curves of generator and discriminator on OPT_Aircraft -7 dataset over training iteration. (**b**) Loss curves of generator and discriminator on MNIST dataset and Fashion-MNIST datasets over training iteration.

Figure 6 provided the loss values of the generator and discriminator in AWCA-GAN during training. Overall, the loss values of AWCA-GAN were stable on three datasets. Especially, about 5000 iterations of OPT _Aircraft-7 dataset, about 2000 iterations of MNIST dataset, and about 4000 iterations of Fashion-MNIST dataset, the upper and lower boundaries of the loss function began to narrow. After convergence, the discriminator and generator trained steadily.

### Evaluation of AWCA-GAN with baseline GAN models

**Figure 7:**
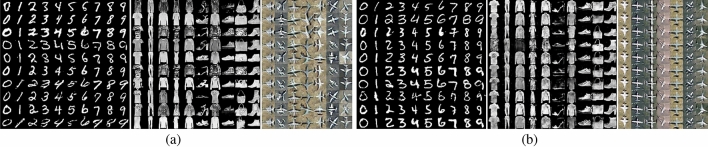
Visual comparison of generated samples on three datasets. (**a**) CGAN^9^. (**b**) ACGAN^10^. From left to right, they are generative samples of MNIST, Fashion-MNIST, and OPT_Aircraft-7 datasets.

To verify the effectiveness of the AWCA-GAN algorithm, we compared it with the current advanced conditional generative models, including CGAN^9^, ACGAN^10^, and SAGAN^21^. In this experiment, the input parameters would affect the performance of the generated model. For fair comparison, we selected the optimal parameters of the author. The visualisation results of CGAN, ACGAN are depicted from left to right in Fig. 7. Each column in each block represented the experimental results using the same datasets, namely MNIST, Fashion-MNIST, and OPT_Aircraft-7.

As illustrated in the visualisation results of the two models on three datasets in Figs. 5 and 7, the generated images from AWAN-GAN come out more distinct and more delicate than that of CGAN and ACGAN. It is the same as expected in our experiment in that the loss value is more stable in the transmission process resulting in higher image quality. In addition, the diversiform results proved the AWCBNA to solve subclass feature entanglement better. The ARSA block could analyse the long-range correlation of feature regions better. Similarly, we observed AWAN-GAN results on MNIST and Fashion-MNIST datasets that synthesise high visual fidelity data.

To further verify the excellence of AWCA-GAN, we calculated the FID score on MNIST (300 epochs), Fashion-MNIST (800 epochs), and OPT_aircraft-7 datasets (10,000 epochs). Lower is better. Referring to Table1, in MNIST and Fashion-MNIST datasets, our model achieved impressive visual effects. In the OPT_aircraft-7 dataset, the FID score of AWCA-GAN improved significantly compared with that of the baseline GAN models. We could see from the score that the AWCBNA and ARSA promote effect on AWCA-GAN and improving the simulation results effectively.

**Table 1:** Evaluation of FID values on MNIST, Fashion-MNIST, and OPT_aircraft-7.

Method	CGAN^9^	ACGAN^10^	SAGAN^21^	AWCA-GAN
MNIST	2.88	0.94	0.30	0.27
Fashion-MNIST	12.84	10.67	0.81	0.31
OPT_aircraft-7	38.37	40.23	19.5	7.5

### Ablation analysis of AWCA-GAN

To explore the effects of AWCBNA and ARSA in AWCA-GAN, we carried out several ablation studies by adding AWCBNA and ARSA to different layers on AWCA-GAN’s generator. The detailed experimental results are listed in Table 2. Table 2 compared FID’s score that AWCBNA and ARSA added into different layers. All models trained in three datasets of MNIST (300 epochs), Fashion-MNIST (800 epochs), and OPT_aircraft-7datasets (10,000 epochs).

The AWCBNA block at the low-level data cube (Blk-1) implemented better performance than the middle-to-high level data cube (i.e., Blk-2 Blk-3). The FID of the model ‘AWCA-GAN, Blk-1’ was improved from 0.35 to 0.27 by ‘AWCA-GAN, Blk-3’ on MNIST. The reason for which is that the AWCBNA can untangle the entanglement features better on the initial feature maps and adjust the value of the inter-class-wise features by adaptive weighting category conditional batch normalisation statistics. However, it only played a minor role due to the short channels when modelling dependencies for bigger feature maps (e.g., ResBlk-2, ResBlk-3). In addition, the comparison of AWCA-GAN (5th column of Table 2) and the ablation model without AWCBNA (4th column of Table 2) showed the effectiveness of our AWCBNA.

**Table 2:** Ablation study on datasets of MNIST, Fashion-MNIST, and OPT_aircraft-7, Blk-n means to add AWCBNA after the n-th ResBlk feature maps, and the best FID report.

AWCBNA	No	No	No	Blk-1	Blk-2	Blk-3	Blk-1
ARSA	No	SA	$\surd$	$\surd$	$\surd$	$\surd$	No
MNIST	0.88	0.54	0.47	0.27	0.30	0.35	0.40
Fashion-MNIST	1.35	1.13	0.76	0.31	0.37	0.42	0.54
Aircraft-7	23.8	21.4	19.3	7.5	8.1	8.8	14.8

Based on the ablation network, we performed another experiment to inspect the effect of ARSA. AWCA-GAN with the ARSA achieved better performance (4th column of Table 2) than AWCA-GAN without the ARSA (2nd column of Table 2) and FID metrics of AWCA-GAN with the self-attention (3rd column of Table 2) reached 0.47, 0.76, and 19.3 on the three datasets. As described in methods, we added the ARSA block to the ResBlk-3 to catch remote dependencies via asymmetric overall regional operations. Compared with the baseline results, the ARSA demonstrated the effectiveness by establishing asymmetric residual distant portions relationships.

### Examples of latent space interpolation

**Figure 8:**
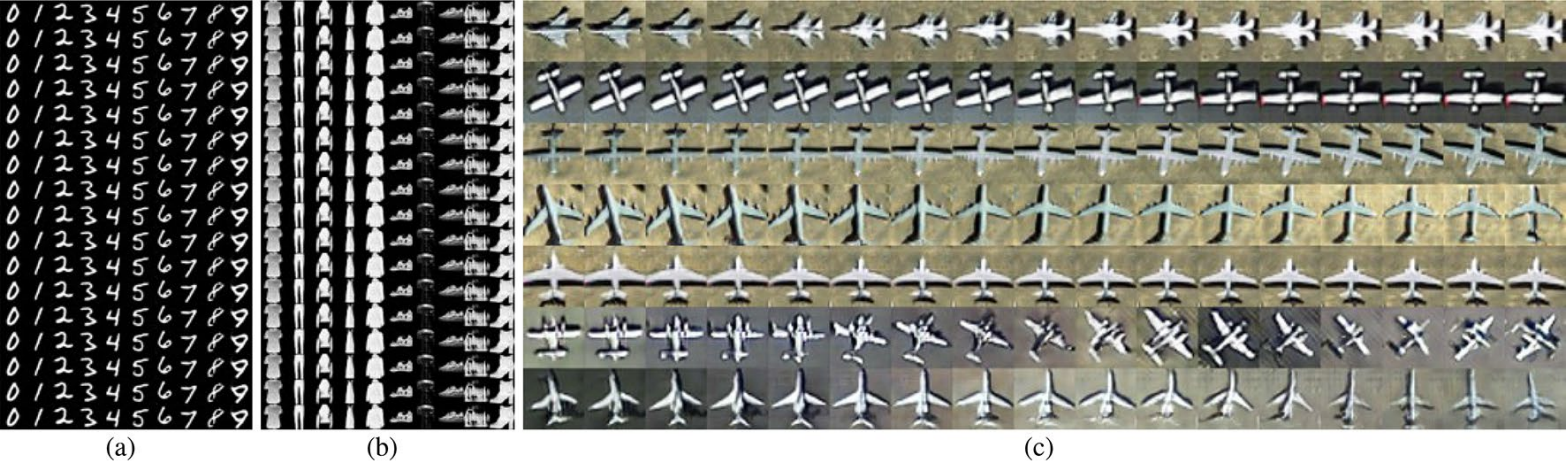
The potential space representation of 17 points between two random vectors $z_{i}$ indicates that the interpolation space has learned smooth and meaningful semantic information transitions based on the same pair of latent variables, allowing nice inter-class interpolation.

To understand the latent space of generated samples in the same classes on MNIST, Fashion-MNIST and Aircraft-7 datasets, we initialized the prior distribution $z \in {\mathbb {R}}^{128} \sim {\mathscr {N}}(0,1)$ in two latent vectors, say $z_{start}$ and $z_{end}$, and interpolated it with the adjustable parameter $\mu _{i}$ to get continuous latent space interpolation vectors $z_{i}=\mu _{i} z_{\text{ start } }+\left( 1-\mu _{i}\right) z_{\text{ end } }, \mu _{i} \in [0,1]$. Figure 8 shows the latent space interpolation results $G\left( z^{*}, y\right)$ on three datasets of trained AWCA-GAN. For OPT_Aircraft-7 in Fig. 8c, we can infer that the AWCA-GAN has learned interesting and pertinent latent space representations. Specifically, at 17 vectors in a series of interpolations $z_{i}$, the evolved potential space has smooth transitions, and each generated image in latent space appears to be an aircraft. In row 2 of Fig. 8c, a flat-wing plane turned from southwest to south gradually. In row 1 of Fig. 8c, a flat-wing plane faced southwest and turned south step by step. These results demonstrated that our generative model could maintain the semantic context in the potential space; thus, confirming that the AWCA-GAN succeeded in controlling the modified region with the user-specifiable embedding coefficients.

### Analysis of embedding results by using GE network

**Figure 9:**
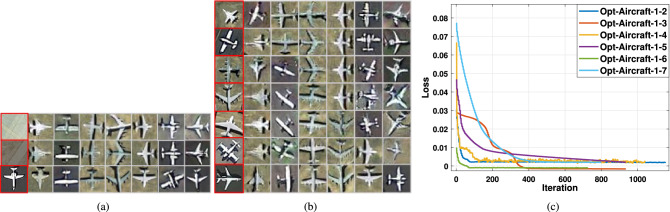
Embedding results and loss value on OPT_Aircraft-7 datasets. (**a**) Each row displays the embedded results from the various ground-truth samples mapped to seven aircraft types, respectively. (**b**) Embedding results on input seven aircraft types samples. The red box represents the ground-truth 32 pixels $\times$ 32 pixels images. The corresponding rows denote that converted to other classes in turn. (**c**) Loss values vs. the number of optimisation steps on OPT_Aircraft-7.

The embedding results were computed by GE with several samples $I \in {\mathbb {R}}^{n \times n \times 3}$ and an embedding label *y*. In Fig. 9a, two real RS scenes and one aircraft image were fed into the GE network and then the input samples were presented to the seven classes embedding results on the OPT_Aircraft-7 dataset. It demonstrated that the GE network learns features of the input samples and finds more nuanced latent space representations to engender excellent embedding. For example, in the top row of the dark background and the middle row of the bright scene in Fig. 9a, the embedding aircraft exhibited multiple intensity levels according to the input RS scenes brightness information; thus confirming that the GE network could capture luminance details successfully. In addition, the bottom row in Fig. 9a also demonstrated that the GE network could capture the structural details of input aircraft samples such as direction, lighting, and shadows.

Figure 9b and 9c depicted the embedding results and the loss curve of input RS images on OPT _Aircraft-7. The GE network can learn the intensity of light and the direction of shadows and so on from the input aircraft samples in the process of embedding. The loss function of the OPT_Aircraft-7 dataset converges at around 800 optimisation steps.

**Figure 10:**
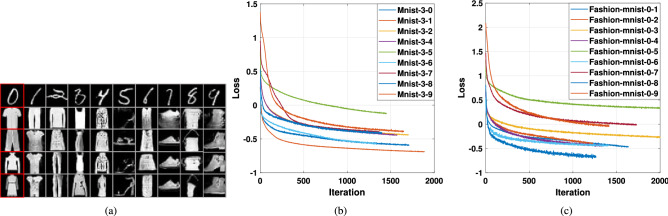
Embedding results and loss value on MNIST and Fashion-MNIST datasets. (**a**) The red frames represent the ground-truth 32 pixels $\times$ 32 pixels images *x*, and columns 2 to 10 represent the embedding results, $G\left( z^{*}, y\right)$, by the GE with various class labels *y*. (**b**) Embedding loss values vs. the number of optimisation steps on MNIST. (**c**) Embedding loss values vs. the number of optimisation steps on Fashion-MNIST datasets.

Furthermore, we performed embedding experiments on MNIST and Fashion-MNIST datasets. Figure 10 showed the five embedding examples and loss values. Figure 10a presented the embedding result of the number 0 from 1 to 9 and four embedding results of Fashion-MNIST samples. Interestingly, the converted 1 to 9 capture narrow texture features from the input digit 0 samples successfully, and it appears to be written by the same person (e.g., row 1 in Fig. 10a). Similarly, the converted clothes have a similar skinniness to the input clothes samples (e.g., row 2,4, column 1,4 in Fig. 10a). This phenomenon revealed that the GE network has good generalisation and expression power. The well-converged train history of GE was shown in Fig. 10b and c. The loss function of the MNIST dataset converged at approximately 800 optimisation steps, and the Fashion-MNIST dataset converged at approximately 1200 optimisation steps.

### Simulation results of ATSS-1

**Figure 11:**
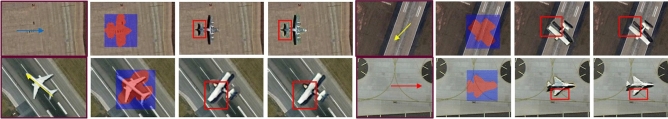
Examples of ATSS-1 vs pixel collage (naive copy-and-paste). The purple border presents the real scenes. The arrows represent the relevant parameters of the ATSS-1, including geographical coordinates, embedding type, direction, and resolution. The red-coloured region and blue-coloured region in columns 2,6 present the blending mask, where red represents the collage area and blue represents areas that are not collaged. Column 3,7 represent pixel collage. Columns 4,8 depict the Poisson collage.

Because trained GE and AWCA-GAN models cannot disentangle objects from the ground-truth images, naively pasting the generated clip to the target image can produce artifacts in the region surrounding the object of interest. We cleaned up these artifacts with Poisson blending to the region of interest. Figure 11 depicted four examples of the application of ATSS-1 and direct pixel collage comparison. We succeeded in making semantic modifications like “changing aircraft type” (Row 2, column 1 in Fig. 11). Column 4,8 in Fig. 11, the embedding samples fused into the real RS scene using Poisson fusion, which appears naturally in the final fused results by changing the intensity of the feature space. The direct collage method (column 3,7) exposed abrupt boundary information between the aircraft and the background by comparison. It was found that the ATSS-1 could synthesise simulated images with high visual and satisfactory visual effects.

## Discussion

We proposed a novel simulation system, based on an improved conditional GAN to achieve different RS images’ 4V modelling. The ATSS-1 put forward a new exploration idea and solved three existing problems. First, for the issue of feature entanglement between the sub-classes using simple GAN to generate different sub-class images, we proposed an AWCBNA module in AWCA-GAN. The AWCBNA reassigned the category-wise responses adaptively by mining the inter-class-wise feature statistics. Second, we improved the self-attention module ARSA for more detailed texture modelling. In ARSA, an asymmetric self-attention map was formed in the spatial channel to achieve better feature representation. Third, we introduced the GE network in the front-end of AWCA-GAN to optimise the mapping from the input RS scene to a latent space of the generated target. In this manner, we preliminarily realised that the generated targets could change with RS scenes such as brightness, shadow, and direction. Particularly, we collected and published an aircraft dataset type of OPT_Aircraft-7, which was successfully applied in our simulation experiment and its effectiveness was demonstrated. In addition, we also performed ablation experiments on the proposed modules and compared them with the state-of-art methods, showing superior results. Enabled by the disentanglement and the fine space mapping properties, ATSS-1 realised 4V modelling on a given RS dataset.

Overall, this paper preliminarily explores a 4V modelling method for a variety of RS aircraft. Our generative model joint embedding network demonstrates excellent development potential for the M&S of RS. As the first attempt, we mainly conducted simulation analysis on the OPT_aircraft-V1.0 dataset and configured a simulation unit at a low resolution of 32*32 pixels. Future research will improve the resolution of simulation targets to 64 * 64 pixels or 128 * 128 pixels. At the same time, we will expand the RS target dataset to realise different types of target simulation, such as buildings, traffic vehicles, and rivers to provide a rich and influential data foundation for high-resolution RS.

## Data Availability

The OPT-Aircraft_V1.0 datasets is available online at http://www.geodoi.ac.cn/WebEn/doi.aspx?Id=1493.
